# Resolution of Superimposed Linear Psoriasis With Bimekizumab After Resistance to Several Biologics

**DOI:** 10.7759/cureus.84452

**Published:** 2025-05-20

**Authors:** Kazuki Yatsuzuka, Jun Muto, Satoshi Yoshida, Ken Shiraishi, Yasuhiro Fujisawa

**Affiliations:** 1 Department of Dermatology, Ehime University Graduate School of Medicine, Toon, JPN; 2 Department of Dermatology, Ehime University Graduate School of Medicine, Ehime, JPN

**Keywords:** bimekizumab, biologics, interleukin-17, psoriasis, superimposed linear psoriasis

## Abstract

Superimposed linear psoriasis is an uncommon form of psoriasis characterized by linear skin lesions aligned with Blaschko’s lines, appearing in conjunction with typical psoriatic plaques. This variant is believed to involve cutaneous mosaicism and often shows differential treatment responses between linear and conventional lesions. We present the first case of superimposed linear psoriasis that was resistant to several biologic agents but responded favorably to bimekizumab, a monoclonal antibody targeting both interleukin (IL)-17A and IL-17F.

A 33-year-old Japanese male with widespread plaque psoriasis developed a persistent, itchy linear lesion on his right thigh despite prior treatment with cyclosporine and multiple biologics, including ustekinumab, guselkumab, and risankizumab. While generalized psoriasis improved with IL-23 inhibitors, the linear component remained unaffected. Upon initiating bimekizumab at age 45, the patient achieved remission of classical plaque psoriasis within four weeks, and the linear lesion showed marked improvement over a year. This case highlights the potential utility of dual IL-17A/F inhibition in treating this rare and difficult-to-manage psoriasis subtype and suggests a need for further clinical evaluation in larger patient populations.

## Introduction

Blaschko linear psoriasis is an uncommon variant of psoriasis characterized by lesions that follow the lines of Blaschko, which represent pathways of embryonic cell migration and are invisible under normal conditions but become apparent in certain skin disorders. This distinct distribution pattern suggests a role for cutaneous mosaicism (where a genetic mutation affects only a subset of skin cells) in its pathogenesis. Clinically, Blaschko linear psoriasis is categorized into two types based on the pattern of presentation [1]. Type I (isolated type) manifests solely as linear lesions without the presence of classical psoriasis forms such as plaque or guttate psoriasis. In contrast, type II, also referred to as superimposed linear psoriasis, occurs in the context of more widespread conventional psoriatic involvement [1]. In this subtype, linear lesions may become more apparent following treatment-induced regression of background plaques. Type II has been reported to occur more frequently in male patients [1]. In superimposed linear psoriasis, treatment responses typically fall into three patterns: linear psoriasis resistant with classical plaques responsive, both responsive, or linear psoriasis responsive, with the first pattern being the most common [1-3]. Here, we report the first case of superimposed linear psoriasis effectively treated with bimekizumab after the failure of multiple biologics.

## Case presentation

In 2010, a 33-year-old Japanese man presented with a three-year history of widespread, scaly, erythematous plaques. He had a notable medical history of smoking and spontaneous pneumothorax, and no relevant family history was identified. Initial treatment with topical corticosteroids yielded no significant clinical improvement. Upon examination, the patient exhibited a psoriasis area and severity index (PASI) score of 41.8, with no evidence of joint involvement, leading to a diagnosis of psoriasis vulgaris. Systemic therapy with cyclosporine was initiated, resulting in substantial improvement of the generalized skin lesions. However, he subsequently developed intensely pruritic, scaly, erythematous plaques and papules with excoriations localized to his right thigh, distributed along Blaschko’s lines. At that point, superimposed linear psoriasis was suspected, and the patient was continued on cyclosporine in conjunction with topical corticosteroids. Although his PASI score improved significantly to 6.3, the linear thigh lesion remained unresponsive. The differential diagnosis at that time included both superimposed linear psoriasis and the possibility of psoriasis and concomitant inflammatory linear verrucous epidermal nevus [4], a condition that, while typically presenting at birth or in early childhood, can occasionally develop in adulthood.

**Figure 1:**
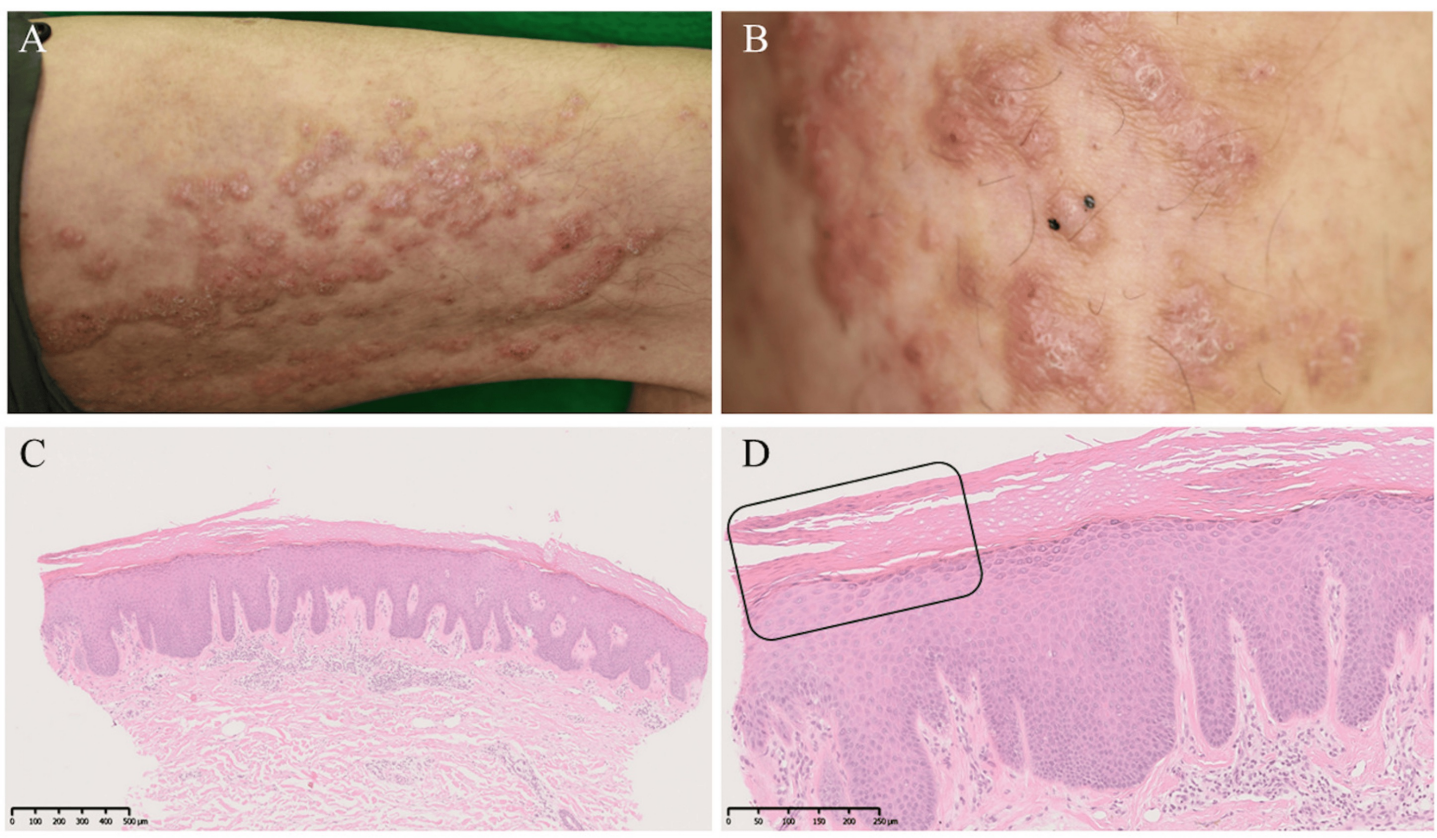
Cutaneous and histological findings (hematoxylin and eosin stain) of the linear lesion A, B: Pruritic, scaly erythematous plaques and papules with excoriations on the right thigh along Blaschko’s lines; B: A skin biopsy from the right thigh; C: Hyperkeratosis and psoriasiform regular acanthosis seen on low-power magnification; D: Parakeratosis with mild underlying hypogranulosis and lymphocytic infiltration around the superficial dermal vessels (rectangular box) seen on high-power magnification The scale bars are 500 µm in image C and 250 µm in image D.

In 2011, the patient’s treatment regimen was changed to ustekinumab (45 mg); however, his PASI score remained stable at 6-8, with persistent involvement of the thigh lesion (Figure 1A). A skin biopsy obtained in 2014 from the affected area (Figure 1B) revealed histopathologic findings of hyperkeratosis, parakeratosis with mild underlying hypogranulosis, psoriasiform regular acanthosis, and superficial perivascular lymphocytic infiltration (Figures 1C-1D), findings consistent with chronic, treated psoriasis-supporting the diagnosis of superimposed linear psoriasis.

**Figure 2:**
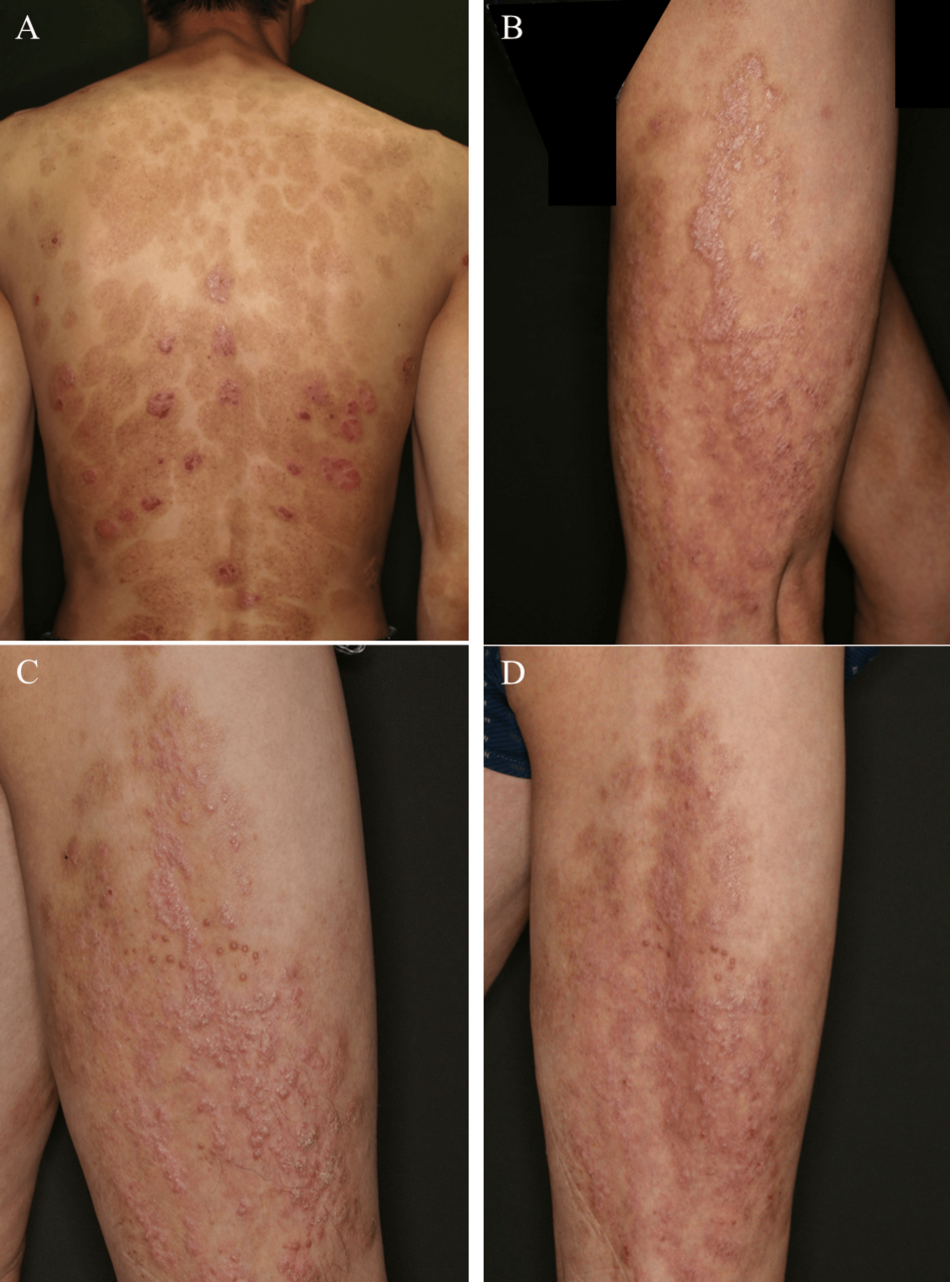
Clinical course of cutaneous manifestations in the present case A: After increasing the ustekinumab dose to 90 mg, the PASI score dropped by 4 to 5, but the linear lesion remained unchanged; B and C: Following risankizumab initiation, the linear lesion remained unaffected; D: One year after the initiation of bimekizumab therapy, the linear lesion demonstrated marked improvement in thickness and hyperkeratosis PASI: Psoriasis area and severity index

Ustekinumab was subsequently increased to 90 mg in 2015, resulting in a modest reduction of the PASI score to 4-5. However, the linear lesion remained refractory, despite concomitant use of a topical combination of corticosteroids and vitamin D3 analogues (Figures 2A-2B). The initiation of guselkumab in 2019 failed to yield any clinical improvement. In 2020, risankizumab was introduced, leading to a marked improvement in generalized psoriasis with a PASI score reduction to 1.6 within eight months; however, the linear lesion persisted without significant change (Figure 2C). Given the patient’s strong desire for complete lesion clearance, bimekizumab therapy was initiated at age 45, three years after starting risankizumab. Remarkably, complete PASI clearance was achieved within four weeks of treatment initiation, and the previously recalcitrant linear lesion began to show clinical improvement. After one year of continued bimekizumab therapy, the linear lesion demonstrated marked improvement in both thickness and hyperkeratosis, with a notable decrease in pruritus (Figure 2D), indicating substantial therapeutic benefit. Notably, no apparent drug-related adverse events were observed throughout the treatment course.

## Discussion

In this superimposed linear psoriasis case, classical psoriatic lesions responded to interleukin (IL)-23 inhibitors, with further improvement following dual IL-17A/F inhibition by bimekizumab. In contrast, the linear lesion was resistant to IL-12/23 and IL-23 inhibitors, showing improvement only after treatment with bimekizumab. As mentioned above, this differential treatment response is often observed in superimposed linear psoriasis and is considered a characteristic feature in response to conventional psoriasis therapies [1-3]. However, because superimposed linear psoriasis is an extremely rare subtype of psoriasis, information regarding its responsiveness to biological agents remains very limited. Although the treatment of superimposed linear psoriasis with biologics such as infliximab, adalimumab, etanercept, ustekinumab, secukinumab, and ixekizumab has been reported [1,5], it is often observed that linear psoriasis is resistant to biologics while classical plaques remain sensitive [1]. This difference in treatment response to biologics suggests that linear psoriasis may represent a distinct nevoid variant, biologically separate from classical plaque psoriasis [5].

Onoufriadis et al. performed RNA sequencing on skin samples from two patients with Blaschko linear psoriasis, comparing affected and unaffected areas and cross-referencing the results with public datasets from psoriasis vulgaris [6]. They observed overlapping patterns of inflammation and keratinocyte activity between Blaschko linear psoriasis and psoriasis vulgaris but also detected notable differences, particularly in pathways related to IL-4, IL-13, and IL-36 signaling. These findings point to a distinct disease mechanism in Blaschko linear psoriasis, though confirmation will require studies with larger patient cohorts [6]. Furthermore, while the efficacy of IL-17A inhibitors has been reported in both types [1], their study focused on type I Blaschko linear psoriasis rather than superimposed linear psoriasis, underscoring the need for comprehensive transcriptomic analyses specifically targeting the latter in future research.

To our knowledge, this is the first report of superimposed linear psoriasis refractory to three or more biologics, including IL-23 inhibitors, responding to bimekizumab. Although IL-17A inhibitors were not used in this case, making it difficult to compare the efficacy of IL-17A monotherapy versus IL-17A/F dual inhibition, IL-17 inhibition may represent a rational therapeutic approach for superimposed linear psoriasis. Further studies are warranted.

## Conclusions

This case demonstrates the successful use of bimekizumab in a patient with superimposed linear psoriasis refractory to multiple biologics, including IL-12/23 and IL-23 inhibitors. While classical psoriatic lesions improved with prior therapies, the linear component remained recalcitrant until treatment with bimekizumab, suggesting distinct pathogenic mechanisms. To our knowledge, this is the first report of its kind, supporting the potential efficacy of dual IL-17A/F inhibition in this rare psoriasis subtype. Given the limited treatment options for superimposed linear psoriasis, bimekizumab may offer a promising alternative when other biologics fail. Further investigation in larger cohorts is warranted to validate these findings.
